# Blood lactate levels in 31 female dogs with pyometra

**DOI:** 10.1186/1751-0147-51-2

**Published:** 2009-01-09

**Authors:** Ragnvi Hagman, Bert Jan Reezigt, Hanna Bergström Ledin, Erika Karlstam

**Affiliations:** 1 Department of Clinical Sciences, Division of Small Animals, Faculty of Veterinary Medicine, Swedish University of Agricultural Sciences, Box 7054, SE-75007 Uppsala, Sweden; 2 Blå Stjärnan Small Animal Hospital, Gjutjärnsgatan 4, SE-41707 Gothenburg, Sweden; 3 The Veterinary Clinic Kusthöjden, Kusthöjden 4, SE-87133 Härnösand, Sweden; 4 Department of Pathology and Wildlife Diseases, National Veterinary Institute, SE-75189 Uppsala, Sweden

## Abstract

**Background:**

Canine pyometra is a life-threatening disease common in countries where spaying of dogs is not routinely performed. The disease is associated with endotoxemia, sepsis, systemic inflammatory response syndrome (SIRS) and a 3–4% mortality rate. Blood lactate analysis is clinically valuable in predicting prognosis and survival, evaluating tissue perfusion and treatment response in human and veterinary critical care settings. The aims of the present study were to investigate 1) the blood lactate levels of female dogs with pyometra by a hand-held analyser and 2) if these levels are related with the clinical status or other biochemical or hematological disorders.

**Methods:**

In total 31 female dogs with pyometra admitted for surgical ovariohysterectomy and 16 healthy female control dogs were included in the present study. A complete physical examination including SIRS-status determination was performed. Blood samples for lactate concentrations, hematological and biochemical parameters, acid-base and blood gas analysis and other laboratory parameters were collected and subsequently analysed. The diagnosis pyometra was verified with histopathological examination of the uterus and ovaries. Increased hospitalisation length and presence of SIRS were used as indicators of outcome.

**Results:**

In the pyometra group the median blood lactate level was 1,6 mmol l^-1 ^(range <0.8–2.7 mmol l^-1^). In the control group the median lactate level was 1,2 mmol l^-1 ^(range <0.8–2.1 mmol l^-1^). Of the 31 bitches 19 (61%) fulfilled 2 or more criteria for SIRS at inclusion, 10 bitches (32%) fulfilled 3 of the SIRS criteria whereas none accomplished more than 3 criteria. Lactate levels did not differ significantly between the pyometra and control group, or between the SIRS positive and SIRS negative dogs with pyometra. Increased lactate concentration (>2.5 mmol l^-1^) was demonstrated in one female dog with pyometra (3%), and was not associated with longer hospitalisation or presence of SIRS. Lactate measurement was not indicative of peritonitis. None of the bitches died during or within two months of the hospital stay. The measurements of temperature, heart rate, respiratory rate, percentage bandforms of neutrophilic granulocytes, α_2_-globulins, creatinin, pvCO_2_, TCO_2 _and base excess showed significant differences between the SIRS positive and the SIRS negative pyometra cases.

**Conclusion:**

Increased blood lactate concentrations were demonstrated in 3% (1/31), and SIRS was present in 61% (19/31) of the female dogs with pyometra. Preoperative lactate levels were not related with presence of SIRS or prolonged hospitalisation. Lactate measurement was not indicative of peritonitis. The value of a single and repeated lactate analysis in more severely affected cases remains to be determined.

## Background

Canine pyometra is diagnosed in almost 25% of all entire bitches before they are 10 years old [1]. The disease is characterised by chronic uterine inflammation with bacterial growth of mainly *Escherichia coli (E. coli) *[2]. Clinical symptoms from the genital tract such as purulent vaginal discharge are often accompanied by generalised signs of disease such as depression, polyuria, polydipsia, lethargy, fever and gastro-intestinal disturbances [3]. The disease is associated with endotoxemia, sepsis, systemic inflammatory response syndrome (SIRS) and is potentially life-threatening with a mortality rate of 3–4% [1,4,5].

The clinical value of blood lactate measurement in predicting prognosis and outcome, determining tissue perfusion and evaluating treatment response in human intensive care units has been well documented [6-11]. Both the initial blood lactate concentrations and the duration of hyperlactatemia can be used to predict survival or death in human trauma patients [12,13]. In septic human patients, serum lactate levels have been closely related to severity of illness and metabolic acidosis [14]. In children with sepsis, blood lactate measurement has been reported as an early indicator for survival [15]. Although a single lactate determination is clinically useful, several sequential measurements of lactate have been shown to have the highest prognostic value [10]. Admission and persistent hyperlactatemia has also been associated with nonsurvival in foals [16]. The development of easily portable point-of-care, rapid, cost-effective analysers have increased the value of lactate measurements for intensive care units in both human and veterinary medicine [6,17,18].

The value of blood lactate measurement as a prognostic indicator for survival has been studied in several animal species, including dogs [10]. The preoperative plasma lactate level was a good indicator for survival and for assessment of gastric necrosis in gastric dilatation volvulus (GDV) in dogs. Dogs with GDV and a blood lactate level <6.0 mmol l^-1 ^were likely to survive whereas dogs with lactate levels >6.0 mmol l^-1 ^had about a 50% chance of survival [19]. Lactate measurement was also valuable in the prediction of outcome in a study of dogs with severe babesiosis [20]. Dogs with babesiosis and blood lactate level over 4.44 mmol l^-1 ^had in spite of initial treatments a much worse prognosis than dogs whose lactate levels decreased below 4.44 mmol l^-1 ^within24 h [20]. Hyperlactatemia (>2.4 mmol l^-1^) was demonstrated in over 40% of dogs with an intestinal foreign body [21].

The clinical value of blood lactate measurement in canine pyometra patients has not yet been determined. Lactic acidosis may be induced by hypoperfusion, SIRS, sepsis and septic chock in pyometra. We thus hypothesised that preoperative lactate measurement could be valuable in the diagnosis, prognosis and the optimisation of therapy for female dogs with pyometra.

The aims of the present study were to investigate 1) the blood lactate levels of female dogs with pyometra by a hand-held analyser and 2) if these levels are related with the clinical status or other biochemical or hematological disorders.

## Methods

The study was approved by the Uppsala Animal Ethics committee, Sweden. All dog-owners signed an owner consent form prior to inclusion of their pet.

### Dogs

Thirty-one privately-owned female dogs with the presumptive diagnosis of pyometra were included. All were subsequently treated by ovariohysterectomy at the University Animal Hospital (UAH), Swedish University of Agricultural Studies (SLU), Uppsala, Sweden. The diagnosis was based on case history, physical examination and diagnostic imaging using ultrasonography or radiography or both, to demonstrate an enlarged, fluid-filled uterus. The diagnosis was verified visually during the ovariohysterectomy and confirmed by routine histopathological examination of hematoxylin-eosin stained uterine sections performed by one of the authors (EK) at the Department of Pathology and Wildlife Diseases, National Veterinary Institute (SVA), Uppsala, Sweden, according to Dow (1959) [22]. Bitches diagnosed with pyometra were of 25 different breeds, with a mean weight of 24 kg (range 9–42 kg) and a mean age of 7,1 years (range 1,1–13,1 years). The admitting clinician completed a form specifying core temperature, heart rate (HR), respiratory rate (RR), mucus membrane colour, capillary refill time (CRT), location for pain response at abdominal palpation, hydration status and general attitude at the time of admission. Postoperatively the HR, temperature and RR were recorded daily for as long as the patient was admitted, on a special form from which also the length of hospitalisation was determined. Hospitalisation was considered necessary when complications occurred or when the general state of the animal was affected severely enough to require veterinary monitoring, care and treatments. Prolonged hospitalisation (>2 days) after ovariohysterectomy at the UAH generally occurs only if complications are present or if the general condition of the dog is depressed or if other signs of disease are present. Further information on treatment, complication to treatment and mortality were obtained from the medical records. None of the female dogs had previously been medically treated for pyometra. One female dog with pyometra was euthanized at the owner's request after preliminary diagnosis by physical examination, blood analysis and ultrasonographic examination of the uterus since it was 12 years old and suffered from concurrent disease (severe arthrosis). In that patient ovariohysterectomy was immediately performed after euthanasia with permission of the owner.

Healthy adult intact female dogs that were staff-owned (n = 14) and two client-owned female dogs admitted for spaying (ovariectomy) were included in the control group (n = 16). The control bitches were of 13 different breeds, with a mean weight of 24 kg (range 10–34 kg) and mean age of 5,5 years (range 1,2–8,3 years). A history questionnaire was completed to confirm that the owners considered the bitch healthy for at least six months prior to the examination. Complete physical examination was performed and documented by the admitting veterinary surgeon that also completed the form about core temperature, HR, RR, mucus membrane colour, CRT, location of any pain response at abdominal palpation, hydration status and general attitude. None of the control bitches had previously been medically treated for pyometra, nor obtained any uterine disease at least one year after participation in the study, as determined by telephone contact with the owner. During surgery no uterine abnormalities were detected on the two bitches admitted for spaying.

### Sample collection and analysis

Blood samples for biochemical and hematological analysis were obtained from the distal cephalic vein after physical examination (control group) and immediately before surgery (pyometra group) into EDTA, sodium-heparinised, citrate and non-additive vacutainer tubes (Becton-Dickinson, Stockholm, Sweden). After filling of the blood collection tubes and release of the stasis, a drop of whole blood without preservative was analysed by the lactate analyser Lactate Pro^® ^LT-1710 (Arkray Inc., Kyoto, Japan), according to the manufacturer. If the lactate level was >2 mmol l^-1 ^another sample was obtained within the next 6 h, and the analysis repeated every 6 h until the levels were <2 mmol l^-1^. The following parameters were analysed in the heparinised venous blood with the hand-held analyser i-STAT^® ^(Abbott Laboratories, Abbott Park, Illinois, USA): pH, partial pressures of carbon dioxide (pvCO_2_, kPa) and oxygen (pvO_2_, kPa), base excess (BE.B, mmol l^-1^), bicarbonate (HCO_3_, mmol l^-1^), total carbon dioxide (TCO_2_, mEq l^-1^), oxygen saturation (sO_2_, %), sodium (Na, mmol l^-1^), potassium (K, mmol l^-1^), ionized calcium (iCa, mmol l^-1^), glucose (Glu, mmol l^-1^) and packed cell volume (PCV, %). Hemoglobin (Hb, g l^-1^) was calculated from PCV. All other biochemical and hematological analyses were performed at the Clinical laboratory, UAH, SLU, Uppsala, Sweden using routine methods. The hematological analyses performed were total leukocyte count (WBC), differential count and morphological evaluation of blood smears. The serum analyses performed were the enzymes alanine aminotransferase (ALAT) and alkaline phosphatase (AP). Urea, cholesterol and total protein were also determined. Serum proteins were separated on agarose gel into albumin, α_1_-, α_2_-, β_1_-, β_2_- and γ-globulins.

### Determination of systemic inflammatory response syndrome (SIRS)

A patient was determined as SIRS positive if two or more of the following criteria were met: RR >20 min^-1^; HR>120 beats min^-1^; WBC<6 or >16 (×10^9 ^l^-1^) or PBN>3%; temp <38.1 or >39.2°C [23]. By using selected criteria a sensitivity (ability to correctly diagnose presence of SIRS in a SIRS positive dog) of 97% and a specificity (ability to correctly diagnose a SIRS negative dog as SIRS negative) of 64% was accomplished [23]. Other criteria for SIRS-determination in dogs are associated with lower sensitivity [23-25]

### Statistical analysis

Statistical analysis was performed with the programme Minitab^® ^for Windows version 15 (Minitab Inc., Pennsylvania, USA) and SAS version 9,1 (SAS Institute Inc., Cary, N. C, USA). Values below detection limit, which in the data set occurred for lactate, BN, segmented neutrophilic granulocytes, eosinophilic granulocytes and basophilic granulocytes were set to zero in the calculations. Median lactate levels in the pyometra group and the control group were compared using a Mann-Whitney U-test. Two-sample t-tests were used to evaluate differences in physical examination findings, hematological and biochemical data, between the pyometra and the control group and also between the SIRS positive and SIRS negative cases within the pyometra group. Fisher's exact test was used to test for differences in lactate levels in SIRS-positive and SIRS negative cases within the pyometra group. Lactate levels were determined as a) within the considered normal limits (e.g. <2.5 mmol l^-1^) and b) above the normal limits. Covariance analysis was performed to test for associations between hospitalisation lengths, SIRS status and lactate limit. Significance was accepted at p < 0.05 for all statistical tests used.

## Results

### Lactate concentrations, hematological and serum biochemistry data

Hematological and serum biochemistry data from the female dogs with pyometra and the healthy control dogs are presented in Table 1 and 2. In the pyometra group the lactate levels ranged between <0.8–2.7 mmol l^-1^, with a median level of 1.6 mmol l^-1^. In the 16 control bitches the lactate levels ranged between <0.8–2.1 mmol l^-1^, with a median level of 1.2 mmol l^-1^. In two bitches in the control group and in one pyometra case, the lactate level was below the detection limit of the analyser (<0.8 mmol l^-1^). Lactate levels did not differ significantly between the control group and the pyometra group (p > 0.23). The lactate levels in the pyometra group did not differ significantly between SIRS positive and SIRS negative cases (Fisher's exact test, p = 0.839). In the one pyometra case where the lactate level was 2.7 mmol l^-1^, a repeated lactate measurement 6 hours later showed a level of 0.8 mmol l^-1^. There was no association with increased hospitalisation (>2 d) or SIRS and a second lactate measurement. On manual differential count of blood smears, toxic neutrophils were present in 25% of the female dogs with pyometra but none of the control dogs.

**Table 1:** Physical examination, hematological and serum biochemical parameters from 31 female dogs with pyometra and 16 healthy control dogs.

	Pyometra dogs		Control dogs		
			
	mean ± SD (range)	n	mean ± SD (range)	n	p-value (t-test)
Temperature (°C)	39.0 ± 0.7 (37.0–40.4)	31	38.5 ± 0.4 (38.1–39.3)	16	0.023*
Heart rate (min^-1^)	110 ± 24 (66–160)	31	93 ± 22 (60–134)	16	0.018*
Respiratory rate (min^-1^)	28 ± 13 (16–60)	29	19 ± 8 (10–40)	16	0.005*
WBC (×10^9 ^l^-1^)	16.8 ± 9.4 (6.4–37.8)	29	8.59 ± 2.00 (5.5–11.9)	16	0.000*
BN (×10^9 ^l^-1^)	1.5 ± 1.6 (0–4.1)	29	0.31 ± 1.17 (0–4.7)	16	0.008*
PBN (%)	11.6 ± 13.5 (0.0–53.0)	29	3.1 ± 11.7 (0–47)	16	0.040*
SN (×10^9 ^l^-1^)	12.2 ± 7.6 (1.4–28.0)	29	4.94 ± 1.79 (0.1–8.7)	16	0.000*
BaN (×10^9 ^l^-1^)	0.01 ± 0.04 (0.00–0.20)	29	0.03 ± 0.05 (0–0.1)	16	0.321
EoN (×10^9 ^l^-1^)	0.25 ± 0.28 (0.0–1.1)	29	0.62 ± 0.42 (0.1–1.3)	16	0.005*
Lymphocytes (10^9 ^l^-1^)	1.95 ± 1.06 (0.0–4.4)	29	2.13 ± 1.12 (0.7–4.5)	16	0.177
Monocytes (×10^9 ^l^-1^)	1.7 ± 1.3 (0.4–6.0)	29	0.55 ± 0.41 (0.2–1.7)	16	0.000*
ALAT (μkat l^-1^)	0.5 ± 0.4 (0.1–1.8)	27	0.5 ± 0.1 (0.4–0.7)	14	0.390
AP (μkat l-^1^)	6.5 ± 6.0 (1.3–32.4)	28	2.5 ± 1.6 (0.9–5.9)	14	0.002*
Urea (mmol l^-1^)	5.0 ± 3.2 (1.7–18.6)	27	6.9 ± 2.5 (3–14)	14	0.041*
Creatinin (μmol l^-1^)	81 ± 22 (47–132)	26	76 ± 13 (55–111)	14	0.539
BA (μmol l^-1^)	6.9 ± 9.2 (0.1–39.5)	25	4.6 ± 2.4 (1.8–8.9)	14	0.266
Total protein (g l^-1^)	62.4 ± 8.7 (41–77)	28	59 ± 5.5 (49–70)	14	0.269
Albumin (g l^-1^)	23 ± 5 (10–30)	27	28 ± 4 (22–36)	14	0.000*
α_1_-globulins (g l^-1^)	2.4 ± 0.7 (1–4)	27	2 ± 1 (1–4)	14	0.547
α_2_-globulins (g l^-1^)	14 ± 3.2 (9–23)	27	11 ± 2 (8–14)	14	0.000*
β_1_-globulins (g l^-1^)	5.7 ± 1.5 (3–8)	28	3 ± 1 (2–5)	14	0.000*
β_2_-globulins (g l^-1^)	9.9 ± 2.0 (6–15)	27	8 ± 2 (5–12)	14	0.016*
γ-globulins (g l^-1^)	8.5 ± 3.8 (4–20)	27	6 ± 2 (3–9)	14	0.010*
Cholesterole (mmol l^-1^)	9.9 ± 2.9 (5.6–17.7)	18	7 ± 2 (4–11)	14	0.004*

WBC = total white blood cell count; BN = Band neutrophilic granulocytes; PBN = percentage band neutrophils; SN = segmented neutrophilic granulocytes; BaN = basophilic granulocytes; EoN = eosiniphilic granulocytes; ALAT = alanine aminotransferase; AP = alkaline phosphatise; BA = Bile acids, globulins= respective fraction on electrophoresis. *Statistically significant difference.

**Table 2:** Results from blood lactate measurement and various laboratory parameters analysed in the pyometra and control groups.

	Pyometra group mean ± SD. (range)	n	Control group mean ± SD. (range)	n	p-value (Mann-Whitney/t-test)	Reference Value^#^
Lactate	1.3 ± 0.56 (<0.8–2.7)	31	1.2 ± 0.46 (<0.8–2.1)	16	0.603	0.9–1.7

pH	7.40 ± 0.04 (7.33–7.51)	27	7.36 ± 0.04 (7.28–7.42)	16	0.006*	7.31–7.41

pvCO_2 _(kPa)	3.98 ± 0.67 (3.03–5.40)	27	5.02 ± 0.72 (3.95–6.15)	16	0.000*	5.47–6.80

pO_2 _(kPa)	9.4 ± 6.3 (4.4–27.1)	26	7.5 ± 3.6 (4.1–19.3)	16	0.293	NA

BE.B (mmol l^-1^)	-5 ± 2 (-9)-0	27	-3 ± 3 (-6)-(+5)	16	0.028*	(-2)-(+3)

HCO_3 _(mmol l^-1^)	18.6 ± 2.6 (15–24.2)	25	21 ± 2 (18.2–24.8)	16	0.001*	21–27

TCO_2 _(mEq l^-1^)	19 ± 3 (15–25)	24	22 ± 2 (19–26)	16	0.001*	17–25

sO_2 _(%)	85 ± 12 (66–100)	23	82 ± 11 (59–99)	16	0.451	95–98

Na (mmol l^-1^)	140 ± 2 (136–152)	27	142 ± 2 (139–144)	16	0.914	138–146

K (mmol l^-1^)	4 ± 0.3 (3.5–4.6)	27	4.1 ± 0.2 (3.6–4.4)	16	0.111	3.5–4.9

iCa (mmol l^-1^)	1.30 ± 0.08 (1.14–1.43)	27	1.33 ± 0.05 (1.24–1.41)	14	0.040*	1.12–1.32

Glu (mmol l^-1^)	5.2 ± 0.9 (4.1–7.4	28	5.1 ± 0.6 (4.0–6.3)	16	0.336	3.6–6.7

PCV (%)	37 ± 8 (22–48)	27	41 ± 5 (31–49)	16	0.003*	38–51

Hb (g/L) (via PCV)	124 ± 28 (75–163)	27	141 ± 18 (105–167)	16	0.003*	120–170

pvCO_2 _= partial pressure of carbon dioxide in the blood (venous), pO_2 _= partial pressure of oxygen in the blood, BE.B = base excess, HCO_3 _= bicarbonate, TCO_2 _= total carbon dioxide, sO_2 _= oxygen saturation, Na = sodium, K = potassium, iCa = ionized calcium, Glu = glucose, PCV = packed cell volume, Hb = hemoglobin. *Statistically significant difference.^# ^= Reference values for dogs from iSTAT^® ^manufacturer. NA = not available.

### Physical examination findings

Selected clinical features of the pyometra group at admission are presented in Table 3. All control dogs were clinically healthy with normal general condition, normal hydration status, no pain on abdominal palpation and normal mucous membranes. The capillary refill time was 1.5–2 s in all dogs. The mean body weight and age did not differ significantly (p = 0.762 and p = 0.067 respectively) between the control group and the pyometra group.

**Table 3:** Number (n) and proportion (%) of the 31 bitches with pyometra that displayed selected clinical features or SIRS on physical examination.

Physical examination finding	n/31 (proportion)
SIRS	19 (61%)
Pain on abdominal palpation	
- *None*	15 (48%)
- *Cranial abdomen*	1 (3%)
- *Caudal abdomen*	4 (13%)
- *Diffuse*	11 (35%)
Dehydration	
- *none*	7 (23%)
- *mild*	17 (55%)
- *moderate*	7 (23%)
- *severe*	0 (0%)
General condition	
- *normal*	6 (19%)
- *slightly depressed*	16 (52%)
- *moderately depressed*	6 (19%)
- *severely depressed*	1 (3%)
Mucous membranes	
- *normal*	26 (84%)
- *pale*	3 (10%)
- *bright red*	1 (3%)
- *icteric*	1 (3%)

### SIRS

By fulfilling 2 or more of the chosen criteria, 19/31 (61%) of the female dogs with pyometra were determined as SIRS positive. In 10/31 (32%) of the dogs in the pyometra group, 3 of the SIRS criteria were achieved, whereas none accomplished more than 3 criteria. All control dogs were SIRS negative. The following laboratory parameters were different between the SIRS positive and SIRS negative group of pyometra patients: HR, RR, temp, PBN, α_2_-globulins, creatinin, pvCO_2_, TCO_2 _and BE.B (Table 4). Presence of SIRS was not associated with increased lactate levels (p = 0.406) or length of hospitalisation (p = 0.701).

**Table 4:** Mean levels, standard deviations and ranges displayed for the laboratory parameters in the pyometra group that were significantly different between the SIRS positive and SIRS negative cases.

	SIRS-positive pyometra cases mean ± SD (range)	n	SIRS-negative pyometra cases mean ± SD (range)	n	p-value (t-test)
Temperature (°C)	39,2 ± 0,8 (38,1–40,4)	19	38,5 ± 0,3 (38,1–39,1)	12	0,002#
Heart rate (beats min^-1^)	120 ± 24 (80–160)	19	94 ± 12 (66–112)	12	0,000#
Respiratory rate (breaths min^-1^)	33 ± 15 (16–60)	17	23 ± 5 (19–35)	12	0,018#
PBN (%)	15,7 ± 15 (0,0–53)	16	4,4 ± 6,9 (0,0–2,0)	9	0,016#
α_2_-globulins (g l-1)	15,1 ± 3,3 (10–23)	17	12,0 ± 1,9 (9–15)	10	0,005
Creatinine (μmol l^-1^)	86 ± 24 (52–132)	17	13 ± 4 (47–89)	9	0,045
pvCO_2 _(kPa)	3,9 ± 0,7 (3,03–5,34)	19	4,5 ± 0,5 (4,0–5,4)	8	0,027
TCO_2 _(kPa)	18,6 ± 2,4 (16,0–23,0)	17	22,1 ± 2,3 (19–25)	7	0,027
BE.B (mmol l^-1^)	-5,6 ± 2 (-9-(-3))	19	-3,0 ± 2 (-6-0)	9	0,007

PBN = percentage band neutrophilic granulocytes; pvCO_2 _= partial pressure of carbon dioxide in venous blood; TCO_2 _= total carbon dioxide; BE.B = base excess; # = SIRS criterion.

### Peritonitis

Of the 31 female dogs with pyometra only 1 had local abdominal peritonitis identified during surgery. In this case, which was SIRS positive and had a hospitalisation length of 3 days, the preoperative lactate level was 1.9 mmol l^-1^.

### Hospitalisation

Of the 16 female dogs with pyometra, 11 had increased hospitalisation length (>2 d), with a mean length of 3.4 d. In the pyometra case with longest hospitalisation (5 d) the initial lactate level was 2,3 mmol l^-1^. In the control group the 2 ovariectomised bitches stayed one and two days at the UAH, respectively. Seven of the pyometra patients were monitored and treated at the intensive care unit previous to surgery.

### Mortality rates

None of dogs died during the hospital stay, but one female dog with pyometra was euthanized before sutrgery due to the owner's request (a 12 year old suffering from severe degenerative hip joint disease). In this case the lactate level was 1,6 mmol l^-1^.

## Discussion

Increased lactate levels, previously defined as >2.4–2.5 mmol l^-1^, was present in 1 of the 31 female dogs with pyometra [21,26,27]. The lactate level in that patient was 2.7 mmol l^-1 ^indicating a decreased tissue perfusion. Tissue hypoxia may have several causes such as hypoperfusion (decreased cardiac output or hypovolemia), anemia (decreased arterial blood oxygen content) or oedema (decreased tissue ability to mobilise oxygen) [28]. The dog's clinical appearance at physical examination did not indicate such findings with only mild dehydration and SIRS negative status. Initial fluid treatments were successful in restoring tissue perfusion as reflected by a blood lactate level of 1.9 mmol l^-1 ^6 h later. Early identification of tissue hypoxia is an advantage for rapid intervention. Not only hypovolemia or oxygen debt but also metabolic conditions such as hepatic malfunction with decreased lactate uptake or diabetic ketoacidosis may induce hyperlactateemia, but no such indications were indicated by the clinical or laboratory findings of this dog [29].

Clinically, blood lactate analysis is not only useful in detecting dogs with decreased tissue perfusion. Lactic acidosis may also reflect sepsis with impaired hepatic lactate extraction and increased lactate production from the spleen or other organs [30]. The finding of a low blood lactate level in a female dog with pyometra indicates that monitoring and care will not necessary have to be intensive, the total treatment costs will be within the standard level and the prognosis is favourable compared with dogs with hyperlactatemia [31]. The risk of anaesthesia and surgery is also lower in dogs with normal lactate levels, which contributes to a relatively good prognosis for surgical treatment [32].

Median and mean blood lactate levels did not differ significantly between the female dogs with pyometra and the healthy control dogs in the present study. These results indicate that tissue perfusion was stabile in most patients in the pyometra group. The maximum lactate concentration in the 16 healthy controls was 2.1 mmol l^-1^. The hand-held analyser used in the present study has previously been shown to have a good precision and to be reliable and accurate in dogs [18]. In two recent studies where the analyser used here was tested, levels of up to 2.9 mmol l^-1 ^and 3.3 mmol l^-1^were demonstrated in jugular vein samples from 30 and 60 healthy dogs, respectively [18,33]. The lactate concentrations measured in the present study were thus all within the range for healthy individuals. The cephalic vein was used for sampling and different sites (the jugular vein or cephalic vein) alter measured lactate levels albeit not at a significant level [26]. Blood stasis technique and degree of patient cooperation may also affect the lactate measurements according to our clinical experience. The hand-held analyser may slightly underestimate the true lactate concentrations especially when measuring lactate levels over 8 mmol l^-1 ^[18]. However, patients with such high lactate levels are more critically ill than the dogs included here. The prognostic value of blood lactate measurements in more severely affected female dogs with pyometra (with higher blood lactate concentrations) still remains to be determined.

We were not able to find any association of increased lactate levels and mortality since none of the bitches died during the post surgical hospital stay or within two months of surgery. One exception was the female dog with pyometra that was euthanized at the owner's request. The euthanasia was in this case not associated with severity of the pyometra, but the presence of concurrent disease. In this case the lactate level was within the reference range for healthy dogs (1.6 mmol l^-1^).

A single preoperative lactate measurement was not indicative of prolonged hospitalisation length. At the UAH, female dogs generally have fully recovered within 1–2 days after spaying and ovariohysterectomy due to pyometra, and the two spayed dogs in the control group were dismissed the day after surgery. Increased lactate levels (>2.5 mmol l^-1^) were not associated with prolonged hospitalisation and the female dog with pyometra with the highest blood lactate level (2.7 mmol l^-1^), was hospitalised for two days. In the pyometra case with longest hospital stay (5 days) the lactate level was 2.3 mmol l^-1^, which is within the reference range for healthy dogs [18,26,33].

In the present study, blood lactate levels above the normal limits were not associated with presence of SIRS in the pyometra patients. There was also no difference in lactate levels between SIRS positive and SIRS negative pyometra cases (Fisher's exact test, p = 0,839). Other studies, using the same criteria, have shown similar (57%) or larger (80%) proportions of SIRS in canine pyometra [34-36]. The identification of SIRS in dogs is important since it has been associated with higher mortality rates [4]. Lower mean lactate levels have been demonstrated in survivors than in nonsurvivors among dogs with SIRS [37]. The selected criteria for determination of SIRS have a specificity of 64 which includes 36% false-positive cases in the SIRS positive group [23]. It is however clinically important to have a high false-positive rate rather than a high false-negative to avoid failing to identify a SIRS-positive patient which could have severe consequences for that dog. Although 61% of the bitches with pyometra in the present study were determined as SIRS positive, the general condition at admission was moderately depressed in six and severely depressed in only one of the female dogs with pyometra, as judged by the veterinary surgeon in charge. It is possible that the lactate analysis would have had indicative value for SIRS determination if more specific SIRS criteria were used. Excluding the criteria used to define SIRS, only the parameters α_2_-globulins, creatinin, pvCO_2_, TCO_2 _and BE.B were significantly different between the SIRS positive and SIRS negative pyometra cases (Table 4).

Peritonitis is a complication associated with canine pyometra, and septic peritonitis is associated with a mortality rate of 68% [38,39]. Here, only one (1%) of the pyometra cases had peritonitis as identified macroscopically during surgery. In this dog, which was SIRS positive and had a hospitalisation length of 3 days, the preoperative lactate level was 1.9 mmol l^-1^. Pyometra and peritonitis will thus not necessarily induce increased lactate levels. Blood lactate analysis alone has previously not been indicative of septic versus non-septic peritoneal effusion in dogs and cats [40]. Likewise were lactate levels not indicative of death or survival in dogs treated for septic peritonitis [31]. Analysis of blood lactate in combination with peritoneal fluid lactate, however, is valuable for indicating outcome in septic peritonitis [40].

An inflammatory response characterised by leukocytosis with a left shift, neutrophilia and monocytosis was apparent in the pyometra group (Table 1). The levels of α_2_-, β_1_-, β_2_-, and γ-globulins were increased and albumin concentrations decreased, reflecting antibody production (immunoglobulins mainly present in β_2_-, and γ-globulin fractions) as well as an acute phase response. Acute phase proteins such as c-reactive protein, serum amyloid A and haptoglobin have been shown to increase in pyometra and are mainly found in the α_2_- and β-globulin fractions [41,42]. Presence of toxic neutrophilic granulocytes, which has previously been linked with poorer prognosis, was demonstrated in 8 (25%) of the pyometra cases and none of the control dogs [43]. This finding is possibly due to endotoxemia, which has been associated with pyometra [5,44].

The levels of ALAT, creatinin and urea did not differ significantly between the two groups, demonstrating a normal kidney- and liver function and lack of hepatocellular damage in the most dogs. In the pyometra group the levels of AP and cholesterol were significantly increased, most likely reflecting intrahepatic cholestasis [45]. Bile acid concentrations were higher in the pyometra group compared with the control group, but the difference was not statistically significant. Anemia, as reflected by low Hb in the pyometra group, may be caused by toxic effects on the bone marrow, decreased erythrocyte viability and loss of erythrocytes to the uterine lumen. An increased iron affinity by the reticulo-endothelial system and a decreased total iron binding capacity is associated with acute and chronic inflammatory diseases and may also induce iron deficiency and subsequent anemia. Blood glucose levels and sodium and chloride concentrations did not differ significantly between the two groups.

In the female dogs with pyometra, the mean pH was higher than in the control dogs, but within the normal range for healthy dogs in both groups. This finding is in accordance with lactate levels mainly within the reference range. In three of the pyometra cases, pH was below 7.34 and in one pyometra case pH was 7.46. In human patients, serum lactate levels are closely related to severity of illness and metabolic acidosis. The in hospital mortality is also higher in patients with lactic acidosis compared with patients with normal lactate levels and pH [14]. The three dogs here with acidosis had hospital stays of 2, 3 and 5 days, respectively.

Both pvCO_2 _and bicarbonate levels were significantly lower in the pyometra group, with mean values below the reference range. Together with the base deficit (negative value for base excess in all but one pyometra case) this indicates a respiratory compensated mild metabolic acidosis. The low pvCO_2 _could also in some cases reflect a primary respiratory alkalosis. Since the majority of cases in the pyometra group did not show acidemia or alkalemia, compensatory mechanisms were sufficient to maintain pH within the normal range or acid-base disturbances were not present at all. The concentrations of iCa and TCO_2 _were significantly lower in the pyometra group, possibly induced by endotoxemia [46].

## Conclusion

Increased blood lactate levels were demonstrated in 3% (1/31) of the female dogs with pyometra. A single preoperative lactate measurement was not indicative of outcome as determined by presence of SIRS or increased hospitalisation. The value of a single or repeated lactate analysis in more severely affected cases of canine pyometra remains to be determined.

## Competing interests

The authors declare that they have no competing interests.

## Authors' contributions

RH participated in the design of the study, performed most statistical analyses, drafted the manuscript and carried out, together with BJR and HBL the recruitment of cases. BJR and HBL participated in the design of the study and in the writing of the manuscript. EK performed the histopathology examinations and participated in the writing of the manuscript. All authors read and approved the final manuscript.
